# Assassin bugs enhance prey capture with a sticky resin

**DOI:** 10.1098/rsbl.2022.0608

**Published:** 2023-04-26

**Authors:** Fernando G. Soley, Marie E. Herberstein

**Affiliations:** School of Natural Sciences, Macquarie University, Sydney, NSW 2109, Australia

**Keywords:** stereotypic tool-use, spinifex grass, *Triodia*, Reduviidae, *Gorareduvius*

## Abstract

Tool-use in animals is a complex and rare phenomenon, particularly in insects. Tool-use in assassin bugs has been suggested as several species apply adhesive plant resins to their body, which has been hypothesized to function in enhancing prey capture. Here, we staged predatory interactions of resin-deprived and resin-equipped assassin bugs (*Gorareduvius* sp.) and discovered that applying resin as a tool conveys a clear predatory advantage to the assassin bugs. *Gorareduvius* sp. can thus be considered a tool-user, and since this behaviour was present in all individuals, including newly hatched nymphs, tool-use can be considered to be stereotyped. Our study, along with others, suggests that, when compared with other insects, tool-use is disproportionately common within the assassin bugs.

## 1. Introduction

Tool-use is a relatively rare phenomenon in animals, despite its potential usefulness to solving ecological challenges [1–3]. The rareness of this behaviour appears to be related to its inherent complexity [2,3]. Tool-use requires the appropriate choice of an environmental item, and its adequate manipulation to achieve a beneficial outcome [3,4]. This behavioural feat was once thought to be accomplished solely by humans, or animals with particular cognitive abilities [5]. Indeed, the most commonly known tool-using examples are from vertebrates (i.e. mammals and birds), but some invertebrates have joined this select group of animals [1,2,6].

A potentially large group of tool-using invertebrates appears to be found within the assassin bugs (Heteroptera: Reduviidae), particularly in the Harpactorinae and Bactrodinae subfamilies. The fact that several species in this group are commonly found covered in plant resins prompted taxonomists to refer to them collectively as ‘resin bugs’ or ‘sticky bugs’ [7–9]. While for most of these cases, the resin constitutes an environmental item that carries a potential beneficial outcome, these bugs are virtually absent from the tool-using literature, perhaps because a clear function for the resin has not yet been established. Some studies suggest that resin could convey a predatory advantage, or when applied to eggs, could offer a protective function [9–11]. As articulated by Zhang & Weirauch [7], evidence in favour of using the resin as a prey-capturing tool requires tests of prey capture efficiency while hunting different prey types, with and without resin. This is not easily performed as assassin bugs can refrain from hunting in the absence of resin [12].

Spiny hummocks of ‘spinifex’ grass (*Triodia* spp.) are a characteristic feature of arid and semi-arid landscapes of Australia, and several species of spinifex produce a sticky resin [13]. In fact, spinifex resin was highly valued as a hafting adhesive for fabricating tools and weapons for hunting by the first humans that reached Australia [13]. We found *Gorareduvius* sp., an undescribed species of resin bug, to be commonly found amidst the spinifex hummocks. Currently, there is only one described species in this genus (i.e. *G. westraliensis* [14]), which is clearly a separate species from the one treated here [15]. We investigated whether spinifex resin is used by this new species, *Gorareduvius* sp.*,* as a tool for hunting prey. If resin is being used as a tool, we predict that bugs covered in resin will have a higher prey capture success when compared with resin-deprived bugs.

## 2. Materials and methods

Behavioural observations and experiments were performed on 2018 at ‘El Questro Station’, in the East Kimberley region of Western Australia (15° 53.6750′S, 128° 07.9860′E), now recognized as part of the Wandjina-Wungur Native Title claim. The groundcover at the particular site where we found the assassin bugs (i.e. ‘Saddleback Ridge’ and its immediate surroundings) was strongly dominated by ‘spinifex grass’ (*Triodia bitextura*). The assassin bugs were usually found standing on these plants and only seldomly they were found on other shrubs or on the ground, but in such cases, the assassin bugs were always adjacent to spinifex plants. Assassin bugs were collected in the field (using 75 ml plastic vials) and transferred to a nearby campsite (30 min away by walking). To mimic natural conditions, observations and experiments were performed inside a tent that was placed at the campsite (see electronic supplementary material). Assassin bugs were kept in a separate tent, also placed in the field.

Interactions between resin-equipped or resin-deprived *Gorareduvius* and two different prey items were staged in artificial arenas consisting of a transparent glass cylinder (8.5 cm diameter, × 10.5 cm height) sealed with a rubber bottom (0.5 cm thick) that sealed its base. A small stick (a 12.7 cm long skewer) was placed inside as a hunting and resting site for the assassin bugs. Prey were introduced from underneath the arena, through a small, semicircular hole (0.6 cm diameter).

The prey items were chosen to represent natural prey items that offer different predatory challenges. Flies (*Musca domestica*) were chosen to represent large (size range 4.8–7.5 mm; mean 5.8 mm), robust prey that can fly and respond quickly to visual stimuli. Ants (*Iridomyrmex* sp.) were chosen to represent small (size range 3.1–4.5 mm; mean 3.9 mm), thin and somewhat slower prey that nonetheless require precise grasping movements. Flies and ants were collected the same day in which experiments were run (2 h maximum prior to experiments) and were kept in separate plastic vials (70 ml). Each assassin bug was sequentially presented with both prey items, while being equipped with resin, and with the resin gently removed (controlling for this handling procedure in the resin-equipped state; see electronic supplementary material). The order of trials was randomized for each individual (i.e. four different conditions in a repeated-measures design).

All data analyses were carried out in R version 4.0.4 [16]. For most analyses, we developed generalized linear mixed effects models with the ‘lme4’ package [17] (see electronic supplementary material). In all cases, mean estimates are followed by 95% confidence intervals. We performed a total of 129 predation trials using 26 adult bugs (15 females and 11 males). Twenty-two assassin bugs (14 females and eight males) completed all four conditions. Specific sample sizes are given for each analysis.

## 3. Results

**Figure 1.**
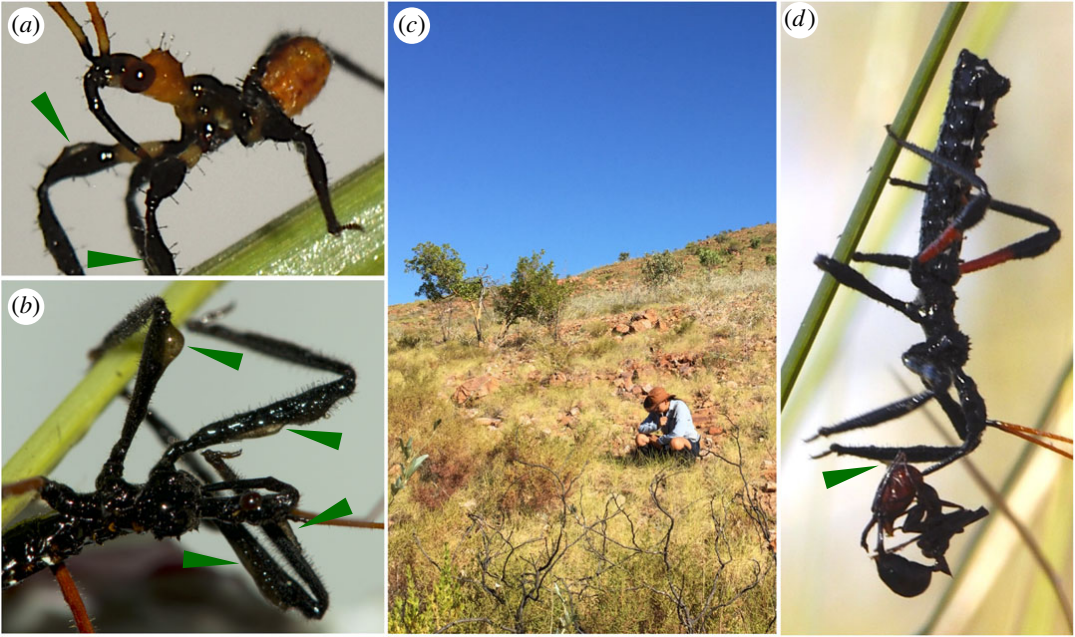
Resin-equipped assassin bugs (*Gorareduvius* sp.) in the East Kimberley region of Western Australia. (*a*) First-instar nymph and (*b*) adult *Gorareduvius* collecting resin from spinifex leaves and applying it onto their forelegs (arrows show resin deposits). (*c*) Spinifex (*Triodia* spp.) hummocks, where assassin bugs were typically found. (*d*) Female adult feeding on an *Odontomachus* ant in the field; arrow points towards a resin deposit.

Field and laboratory observations revealed that *Gorareduvius* ((*n* = 35 females, *n* = 26 males and *n* = 49 nymphs (third–fifth instars)) scraped the resin off the leaves of *Triodia bitextura* and meticulously applied it over the body, particularly onto the forelegs (figure 1; electronic supplementary material, video S1). Resin-scraping and application was also observed in first-instar nymphs reared in isolation (*n* = 15).

**Figure 2.**
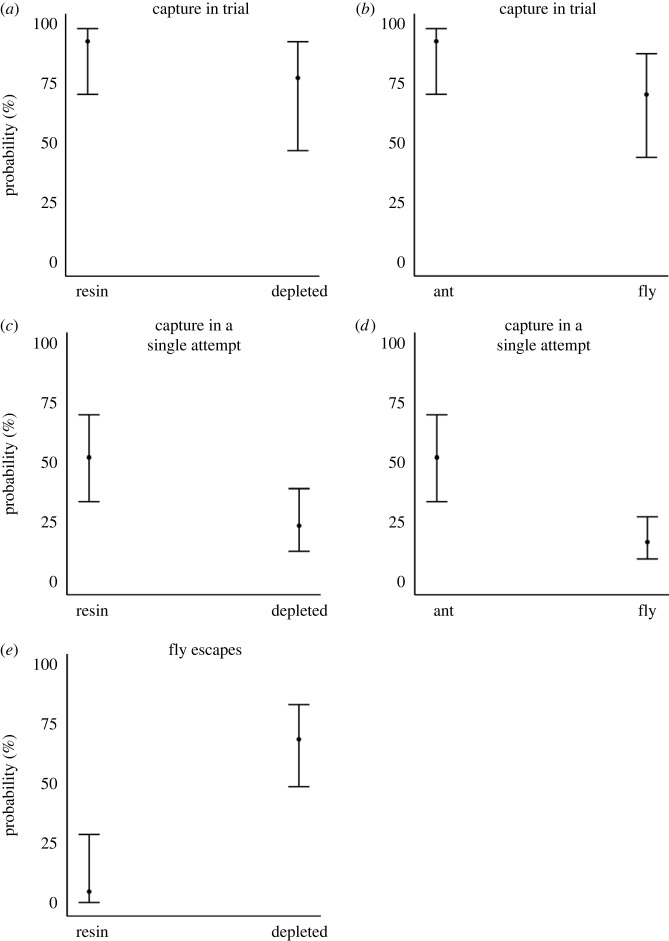
Predatory success of *Gorareduvius* sp. in interactions with ants and flies. Probability of *Gorareduvius* capturing its prey within the allotted time according to (*a*) resin condition, (*b*) prey type; *N* = 65 interactions, 22 assassin bugs. Probability of *Gorareduvius* capturing its prey in each single attack, according to (*c*) resin condition; (*d*) prey type; *N* = 286 attacks, 23 assassin bugs. There was no prey type × resin condition (i.e., treatment) interaction (electronic supplementary material, tables S3 and S4). (*e*) Probability of escape in flies grasped by *Gorareduvius,* according to resin condition (*N* = 45 flies, 26 assassin bugs).

In the experiments, *Gorareduvius* had a 33% (10–56%) higher probability of capturing the prey item within the allotted time frame, if the prey was an ant (table 1 and figure 2). Trials involved one (e.g., if the first attack was successful) or several attacks by the assassin bugs. The success of each individual attack (i.e. each capture attempt of the assassin bug) increased by 27% (12–42%) if the prey was an ant (table 1 and figure 2). In successful trials, *Gorareduvius* required on average 2.6 times (1.8–4.0) the number of attacks to capture a fly (electronic supplementary material, figure S1 and table S1).

**Table 1.** Logistic regression models explaining the influence of prey type and treatment (i.e., resin-equipped or resin-deprived) in the probability of (i) assassin bugs capturing the prey item within the given time frame in the experimental arena (*N* = 65 trials, 22 assassin bugs); (ii) assassin bugs capturing the prey item in each single attack (i.e. each capture attempt) (*N* = 286 attacks, 23 assassin bugs) and (iii) the probability of flies escaping after being grasped by the assassin bugs (*N* = 45 flies, 26 assassin bugs).

model	AICc	dAICc	d.f.	conditional *R*^2^
(i) outcome of trial				
prey type × treatment + bug_id	83.7	2.3	5	0.40
prey type + treatment + bug_id	81.3	0.0	4	0.40
treatment + bug_id	85.6	4.3	3	0.29
prey type + bug_id	83.9	2.6	3	0.25
null	87.7	6.4	2	0.16
(ii) outcome of individual attacks				
prey type × treatment + bug_id	213.7	1.8	5	0.27
prey type + treatment + bug_id	211.9	0.0	4	0.25
prey type + bug_id	221.3	9.3	3	0.16
treatment + bug_id	228.3	16.4	3	0.16
null	239.0	27.1	2	0.02
(iii) outcome of individual attacks in which the assassin bugs grasped the fly				
model	AICc	dAICc	d.f.	Tjur's *R*^2^
treatment × sex	48.0	3.8	4	0.41
treatment + sex	46.5	2.3	3	0.41
treatment	44.2	0.0	2	0.41
sex	65.6	21.3	2	0.00
null	63.4	19.2	1	<0.001

Resin was important for increasing prey capture rates for both types of prey, and there was no interaction between prey type and resin condition. The probability of prey capture in a given trial increased by 26% (21–50%) when *Gorareduvius* were equipped with resin (table 1 and figure 2). Resin-depleted *Gorareduvius* required more than twice (2.3 times; 1.6–3.3 times) the number of attacks to capture their prey (electronic supplementary material, table S1 and figure S1). The probability of a given attack being successful was 20% (7–34%) higher when *Gorareduvius* were equipped with resin (table 1 and figure 2). Once grasped or touched by *Gorareduvius*, flies had much higher chances of escaping (64%; 44–84%) if *Gorareduvius* were depleted of resin (table 1 and figure 2).

## 4. Discussion

Spinifex resin conveyed a predatory advantage to the assassin bugs. This advantage likely derived from the resin's adhesive properties. Prey could still escape after being touched by the assassin bugs, but this was less likely to happen if the assassin bugs were equipped with resin. However, prey never appeared to be fully stuck to the resinous surface of the assassin bugs. Rather, it appears that brief, temporary adhesion, delayed prey responses sufficiently enough for the assassin bugs to grasp and stab their prey (see electronic supplementary material, video).

Widely used definitions of tool-use commonly require that a tool should be detached from its environmental surrounding, and be manipulated by the animal to achieve an adaptive benefit [1,3]. Our study supports that resin can be regarded as a tool used by *Gorareduvius* in the context of predation—the assassin bugs manipulated an environmental item (the resin), by taking it out of its usual context and applying it onto their bodies, thus gaining a selective advantage through improved prey capture. The advantage was maintained across different prey items, suggesting that this tool offers a generalized improvement in prey capture, for both flying and non-flying prey of different sizes.

There are several other species of assassin bugs that collect resins from the environment [7,10,12]. Given the shared sticky nature of these resins, our results add general support for the ‘sticky trap’ hypothesis (*sensu* Zhang & Weirauch [8]), with the possibility of wide-spread tool-use in this group. Several of these species also coat their eggs with resin, which appears to offer a protective function [9,11]. Hence, different selective advantages could have acted separately or in concert, favouring the evolution of tool-use in this group.

Tool-users can be categorized as being either stereotyped (i.e. showing ‘hard-wired’ behaviour) or flexible (e.g. using a tool for various purposes) [2,3]. *Gorareduvius* would thus represent a case of stereotypic tool-use, because the behaviours were consistently expressed in all individuals, in the absence of training or demonstration from conspecifics, including in freshly hatched, isolated nymphs. This also seems to be the case with other assassin bugs [11]. More generally, stereotypic tool-use appears to be the only type of tool-use that has evolved in insects [2,6,18].

It is still unclear why tool-use is more common in certain animal groups than in others [3,5]. The flexible use of tools, like that observed in corvids and apes, appears to be constrained by cognitive abilities [2–4]. In the case of stereotyped tool-use, it is thought that the evolution of this behaviour is constrained by the scarcity of behavioural precursors (i.e. motor routines) that can be easily co-opted into tool-use, and a lack of opportunities for such behaviours to be paired with a useful tool in the appropriate context (i.e. a ‘utility constraint’) [2]. Foliage or tree-dwelling habits are very common among the Harpactorinae and Bactrodinae assassin bugs, so that it is possible that association with plants that produce sticky substances, facilitated the evolution of tool-use in these lineages [10].

The ‘resin bugs’ appear to constitute a large fraction of the assassin bug species [7,10]. Even though a precise estimate cannot be obtained at present, because of a lack of natural history information in this group, tool-use seems to be disproportionately common within the assassin bugs [9,10]. The most recent phylogenetic analysis suggests that tool-use evolved at least three times, and independently in New-World and Old-World lineages of assassin bugs [10]. This represents 19% of the estimated number of independently evolved cases of tool-use in insects, or an independent occurrence ratio (IOR) of tool-use in assassin bugs of 1 : 2267, which is far greater than the general IOR of insects, previously estimated to be 1 : 153 846 [18]. This makes the study of tool-use in assassin bugs a particularly promising case for understanding the ecological and behavioural conditions that facilitated the otherwise unlikely evolution of tool-use.

## Data Availability

All data and code are available and are available from the Dryad Digital Repository: https://doi.org/10.5061/dryad.9p8cz8wmm [19]. Supplementary material is available online [20].
